# Recurrent papillary thyroid carcinoma with brachiocephalic vein tumor thrombus: A rare case report

**DOI:** 10.1016/j.radcr.2026.10.002

**Published:** 2026-10-27

**Authors:** Seyed Zeynab Seyedjavadeyn, Mohammad Shirkhoda, Amirmohsen Jalaeefar, Asma Shafaeipour, Bahman Rasuli

**Affiliations:** a Department of General Surgery, Subdivision of Surgical Oncology, Cancer Institute of Iran, Tehran University of Medical Sciences, Tehran, Iran; b Department of Surgical Oncology, Subdivision of Surgical Oncology, Tehran University of Medical Sciences, Cancer Institute, Tehran, Iran; c Department of General Surgery, Subdivision of Surgical Oncology, Cancer Research Center, Cancer Institute of Iran, Tehran University of Medical Sciences, Tehran, Iran; d Department of Radiology, Arman International Hospital, Jame Jam Imaging Center, Tehran, Iran

**Keywords:** Papillary thyroid carcinoma, Tumor thrombus, Brachiocephalic vein, Thyroid cancer recurrence, Computed tomography

## Abstract

Papillary thyroid carcinoma (PTC) generally has an excellent prognosis and an indolent clinical course. However, major vascular invasion with tumor thrombus formation is exceedingly rare. We report the case of a 63-year-old woman with recurrent PTC presenting five years after total thyroidectomy and radioactive iodine therapy.

Contrast-enhanced CT demonstrated recurrent disease within the thyroidectomy bed, extensive cervical nodal metastases, retrosternal extension, pulmonary nodules, and tumor thrombus involving the left brachiocephalic vein near the SVC junction.

The patient underwent extensive surgical management, including left modified neck dissection, median sternotomy, en bloc resection of recurrent disease, thrombectomy, and primary repair of the brachiocephalic vein. Histopathological examination confirmed recurrent papillary thyroid carcinoma with vascular tumor thrombosis. Postoperatively, the patient received adjuvant radioactive iodine therapy.

At 12-month follow-up, serum thyroglobulin levels were undetectable, and imaging studies demonstrated no evidence of locoregional recurrence. This case highlights a rare and aggressive presentation of recurrent PTC with major venous involvement and demonstrates that satisfactory short-term disease control can be achieved through aggressive multidisciplinary management.

## Introduction

Papillary thyroid carcinoma (PTC) is the most common histological subtype of thyroid malignancy and generally follows an indolent clinical course with an excellent long-term prognosis [1]. Despite favorable outcomes in most patients, locoregional recurrence remains a recognized challenge, occurring in a subset of cases even after apparently successful treatment. Most recurrences develop within the first five years following initial therapy and typically involve cervical lymph nodes or the thyroid bed [2]. While microscopic extrathyroidal extension may be observed in up to one-third of patients, gross invasion of adjacent structures such as the strap muscles, trachea, esophagus, and carotid artery is uncommon, occurring in fewer than 5% of cases [1,3]. Such locally advanced disease has been associated with higher rates of recurrence, distant metastasis, and disease-specific mortality [[3], [4], [5]].

Major vascular invasion with intraluminal tumor thrombus formation is an exceptionally rare manifestation of PTC. Unlike follicular and Hürthle cell carcinomas, which more commonly exhibit hematogenous dissemination, PTC typically spreads through lymphatic pathways [6]. Consequently, invasion of major cervical and mediastinal veins is infrequently encountered. Fewer than 30 cases of PTC associated with tumor thrombus involving the internal jugular vein (IJV), brachiocephalic vein, superior vena cava (SVC), ascending aorta, or right atrium have been reported in the literature to date [1,7]. Furthermore, one large series reported an incidence of tumor thrombus of only 0.116% among 7754 patients undergoing thyroid surgery, highlighting the rarity of this phenomenon [8,9].

Tumor thrombus may develop through direct extension of the primary or recurrent tumor into adjacent venous structures or through intravascular tumor growth following occult vascular invasion [9]. Involvement of the brachiocephalic vein is particularly uncommon, with only a limited number of cases reported in the literature [8,[10], [11], [12], [13]]. Recognition of this rare pattern of disease is clinically important because major venous involvement may significantly influence surgical planning, prognosis, and overall management strategy.

We present a rare case of recurrent papillary thyroid carcinoma with extensive locoregional recurrence involving the thyroidectomy bed, cervical lymph nodes, and left brachiocephalic vein tumor thrombus extending toward the SVC junction. The case illustrates the critical role of cross-sectional imaging in identifying vascular invasion and demonstrates the feasibility of aggressive surgical management in selected patients with advanced recurrent disease.

## Case report

A 63-year-old woman presented to her endocrinologist with a progressively enlarging neck mass and was subsequently referred for surgical evaluation. The patient denied hoarseness, dysphagia, dyspnea, or other compressive symptoms. Her medical history was significant for papillary thyroid carcinoma that had been treated with total thyroidectomy five years earlier. Following surgery, she underwent radioactive iodine (RAI) therapy and was maintained on thyroid hormone replacement therapy. Although she reported adherence to her medication regimen, she acknowledged missing several routine follow-up appointments in recent years. There was no significant family history of thyroid malignancy or other endocrine disorders.

Physical examination revealed a firm palpable mass within the anterior neck at the site of the previous thyroidectomy. Multiple enlarged lymph nodes were palpated within the left cervical region. These lymph nodes were firm, non-tender, and partially fixed to the underlying soft tissues. Direct laryngoscopy demonstrated normal bilateral vocal cord mobility.

Laboratory investigations revealed markedly elevated serum thyroglobulin levels, raising concern for recurrent or persistent disease. Thyroid function testing confirmed adequate thyroid hormone replacement, with thyroid-stimulating hormone (TSH) levels within the therapeutic target range.

Neck ultrasonography demonstrated a heterogeneous hypoechoic mass within the thyroidectomy bed. Multiple suspicious lymph nodes were identified within the left cervical nodal stations (levels II–V), characterized by rounded morphology and loss of the normal fatty hilum, findings concerning for metastatic nodal involvement.

Contrast-enhanced computed tomography (CT) of the neck and upper thorax further characterized the extent of disease. CT demonstrated a 28 × 35 mm recurrent soft-tissue mass within the left thyroidectomy bed with retrosternal extension into the superior mediastinum (Fig. 1). The recurrent lesion invaded the adjacent strap muscles and the left sternocleidomastoid muscle. Multiple enlarged cervical lymph nodes were present within the left neck, consistent with nodal metastatic disease.

**Fig. 1:**
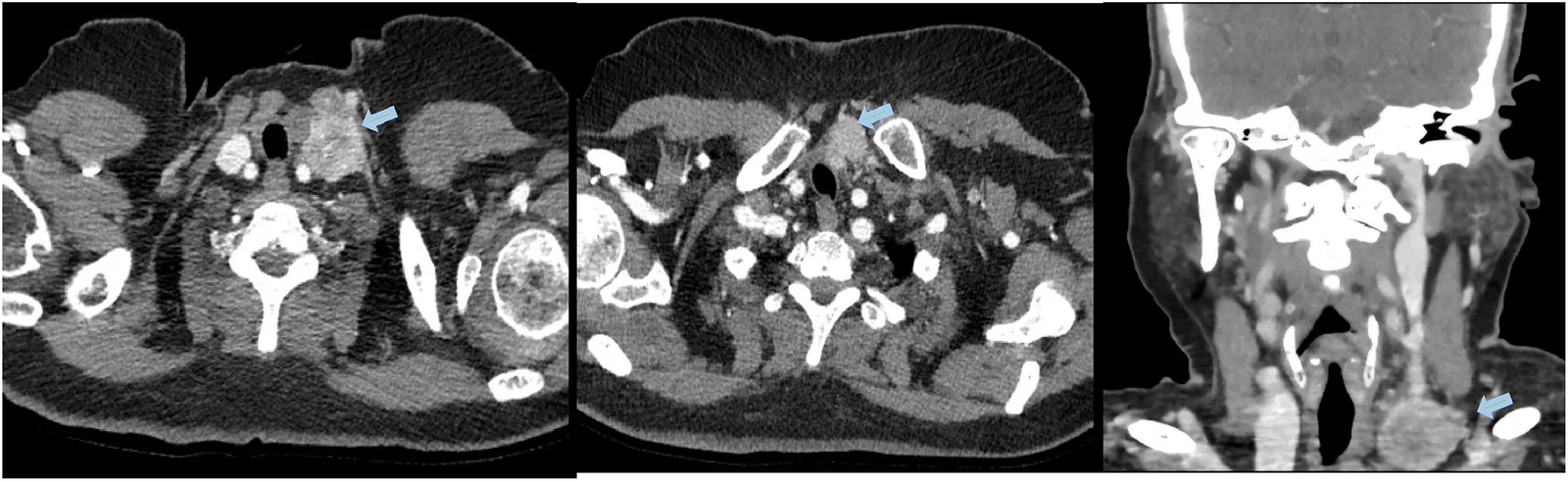
Contrast-enhanced axial CT image of the neck showing a recurrent soft-tissue mass (blue arrows) within the left thyroidectomy bed with local invasion of adjacent strap muscles and retrosternal extension.

Importantly, CT revealed tumor extension into the left brachiocephalic vein with intraluminal tumor thrombus extending toward the junction with the superior vena cava (Fig. 2).

**Fig. 2:**
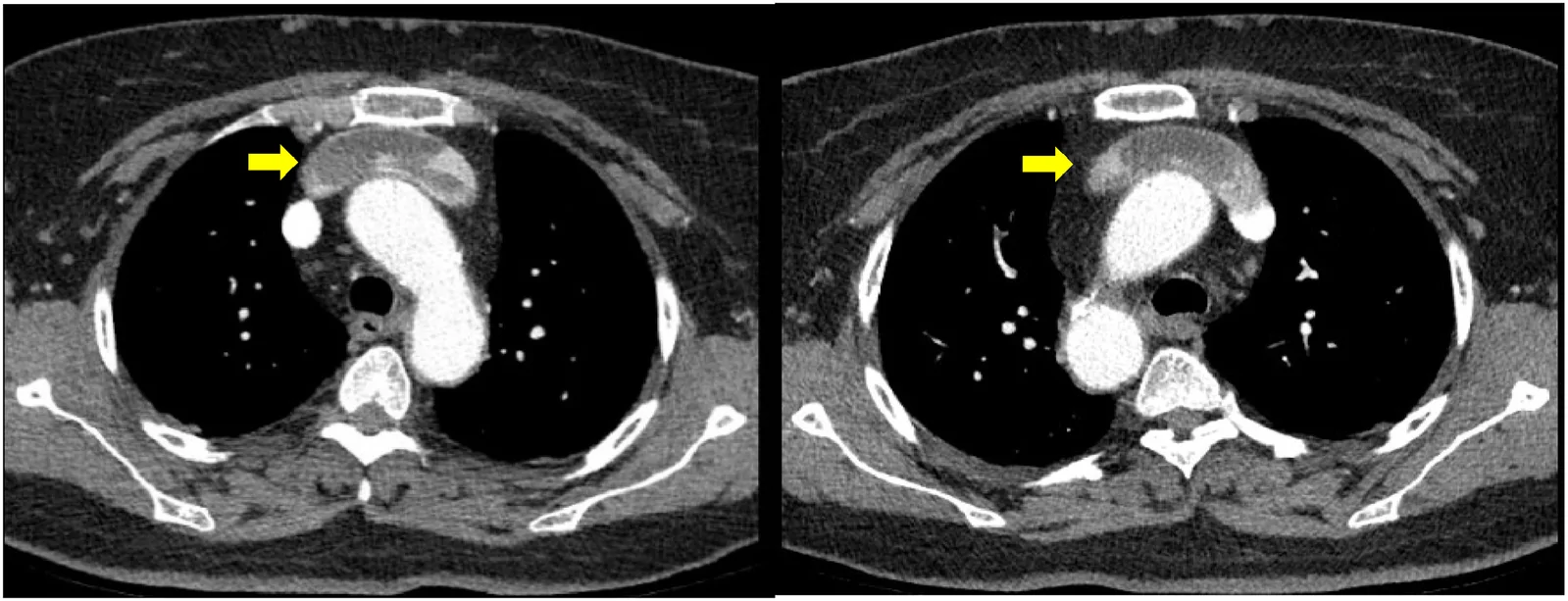
Contrast-enhanced CT of the upper mediastinum showing tumor thrombus (yellow arrows) within the left brachiocephalic vein extending toward the superior vena cava junction.

In addition, two indeterminate pulmonary nodules were identified in the right lung, which were considered suspicious for possible metastatic involvement in the clinical setting of recurrent papillary thyroid carcinoma (Fig. 3).

**Fig. 3:**
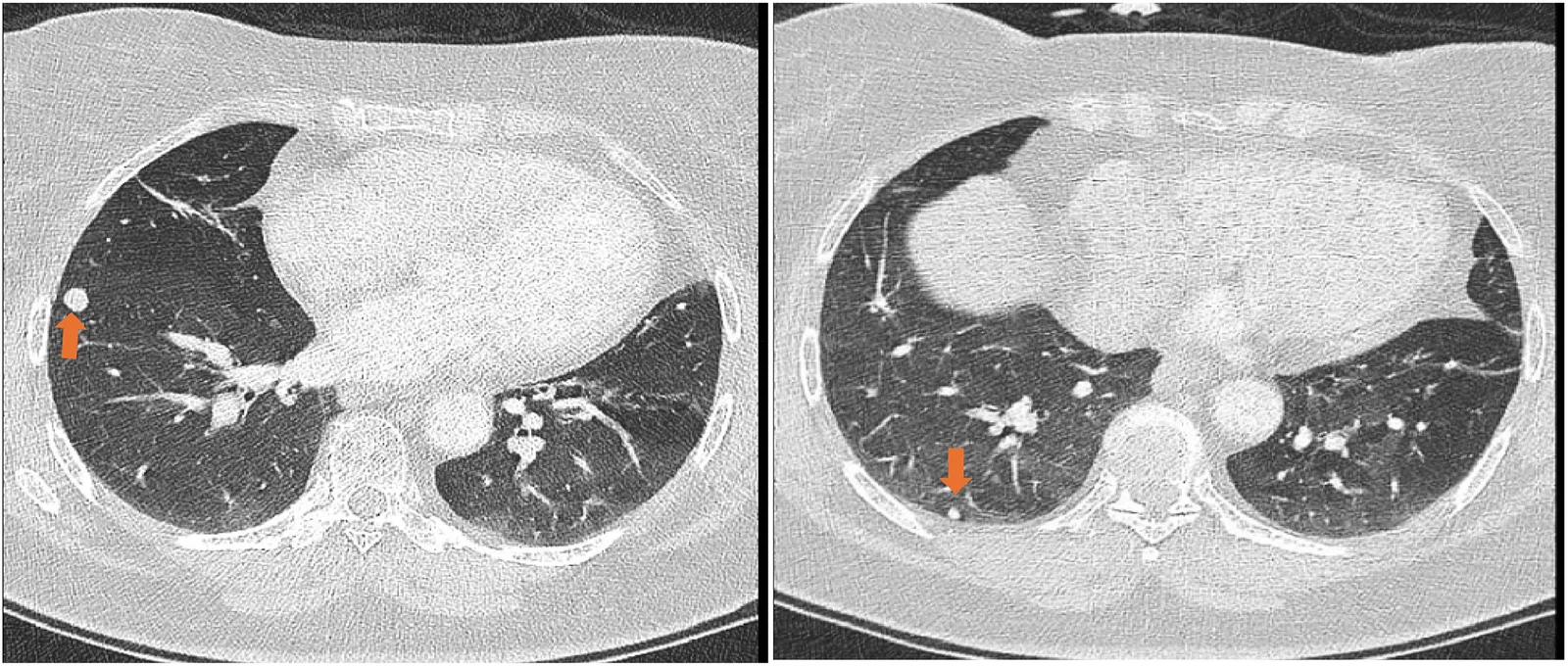
Chest CT demonstrating two small right lower lobe pulmonary nodules (orange arrows). The metastatic nature of these nodules remained unconfirmed.

Fine-needle aspiration cytology (FNAC) of both the thyroid bed mass and enlarged cervical lymph nodes confirmed recurrent papillary thyroid carcinoma.

Following multidisciplinary evaluation, surgical treatment was recommended because of the extensive locoregional recurrence and major venous involvement. Intraoperatively, extensive recurrent disease was identified involving the left thyroid bed, strap muscles, sternocleidomastoid muscle, and left cervical lymphatic compartments. Owing to retrosternal extension and mediastinal involvement, a median sternotomy was required to achieve adequate surgical exposure.

The tumor was found to extend into the left brachiocephalic vein, with tumor thrombus approaching the superior vena cava junction. A left modified neck dissection was performed, followed by en bloc resection of recurrent disease. Venotomy with thrombectomy was subsequently undertaken, and primary repair of the brachiocephalic vein was successfully performed (Fig. 4). Complete macroscopic resection of the locoregional disease was achieved.

**Fig. 4:**
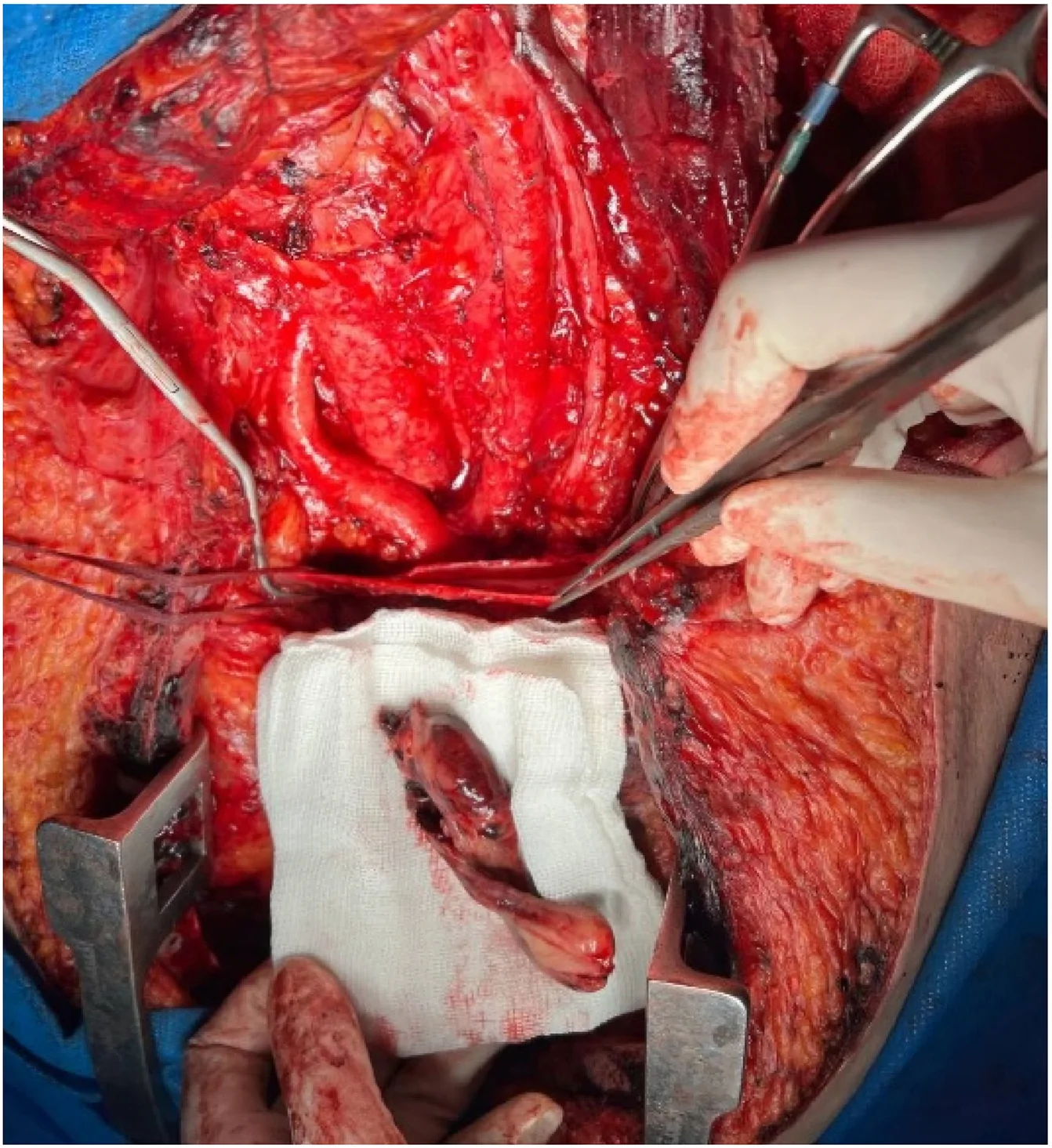
Intraoperative photograph demonstrating tumor thrombus removed from the left brachiocephalic vein following venotomy and thrombectomy.

The patient was transferred to the intensive care unit postoperatively. She was extubated on the first postoperative day and experienced an uneventful recovery. One week after surgery, she was discharged in good clinical condition.

Histopathological examination confirmed recurrent papillary thyroid carcinoma with intravascular tumor thrombosis involving the left brachiocephalic vein. Six weeks following surgery, the patient underwent adjuvant radioactive iodine therapy.

At 12-month follow-up, serum thyroglobulin levels were undetectable. Follow-up imaging demonstrated no evidence of locoregional recurrence, and the patient remained clinically stable.

## Discussion

Papillary thyroid carcinoma (PTC) is the most common histological subtype of thyroid malignancy and is generally associated with an excellent prognosis and long-term survival (1). Although locoregional recurrence may occur, most recurrent lesions are confined to the thyroid bed or cervical lymph nodes and can often be managed successfully with surgery, radioactive iodine therapy, or a combination of both modalities (2). In contrast, extensive local recurrence with invasion of major vascular structures represents an uncommon and clinically challenging manifestation of the disease.

Gross extrathyroidal extension involving adjacent structures such as the strap muscles, trachea, esophagus, and carotid artery occurs in fewer than 5% of patients and is associated with poorer clinical outcomes [1,3]. Vascular invasion resulting in tumor thrombus formation within major cervical or mediastinal veins is even more unusual. One large series reported an incidence of tumor thrombus of only 0.116% among 7754 patients undergoing thyroid surgery, underscoring the rarity of this condition [8,9].

Unlike follicular and Hürthle cell thyroid carcinomas, which more commonly demonstrate hematogenous dissemination, PTC typically spreads through lymphatic channels [6]. Consequently, intraluminal tumor thrombus involving major veins is rarely encountered. Proposed mechanisms include direct extension of tumor into adjacent venous structures or intravascular growth following occult vascular invasion [10]. Because of the rarity of this phenomenon, the natural history and optimal management strategy remain incompletely understood.

Fewer than 30 cases of PTC with tumor thrombus involving major vascular structures, including the internal jugular vein, brachiocephalic vein, superior vena cava, ascending aorta, and right atrium, have been reported in the literature [1,7]. Involvement of the brachiocephalic vein is particularly uncommon and has been documented primarily in isolated case reports [[10], [11], [12], [13]].

Several previously published reports have demonstrated the feasibility of aggressive surgical management in selected patients. Babu et al. described a patient with PTC involving both the internal jugular and brachiocephalic veins who underwent complete resection of the affected venous segments [10]. Similarly, Koike et al. reported successful surgical treatment of a tumor thrombus extending from the brachiocephalic vein into the superior vena cava [11]. Wada et al. described extensive venous involvement requiring superior vena cava reconstruction using autologous tissue, whereas Chung et al. reported direct tumor extension into the innominate vein that was successfully managed through a single operative approach [12,13]. Collectively, these reports suggest that complete surgical excision may offer meaningful disease control when technically feasible.

The present case is noteworthy for several reasons. First, recurrence occurred five years after initial treatment despite total thyroidectomy and radioactive iodine therapy, emphasizing the importance of long-term surveillance in patients with differentiated thyroid carcinoma. Second, the recurrence exhibited an aggressive pattern characterized by involvement of the thyroidectomy bed, cervical lymph node metastases, invasion of adjacent musculature, retrosternal extension, pulmonary nodules suspicious for metastatic disease, and tumor thrombus within the left brachiocephalic vein extending toward the superior vena cava junction. The coexistence of these findings is exceptionally rare and highlights the potential for aggressive biological behavior in a disease that is generally considered indolent.

Cross-sectional imaging plays an important role in the evaluation of recurrent and locally invasive differentiated thyroid carcinoma. Contrast-enhanced CT is particularly useful for delineating the extent of recurrent disease, assessing local invasion and the anatomic relationships between tumor and adjacent vascular, aerodigestive, and mediastinal structures, and identifying disease that may not be adequately characterized by ultrasonography alone. In patients with extensive or invasive recurrent disease, CT can help define vascular involvement, mediastinal extension, and the inferior extent of disease, thereby facilitating assessment of resectability and preoperative surgical planning. In the present case, contrast-enhanced CT accurately demonstrated the recurrent thyroid bed mass, cervical nodal disease, mediastinal extension, and tumor thrombus within the left brachiocephalic vein, findings that were critical for determining the extent of surgery and the need for a multidisciplinary surgical approach [14].

MRI and ^18^F-FDG PET/CT may provide complementary information in selected patients with recurrent or advanced thyroid carcinoma. MRI offers superior soft-tissue contrast and can be useful for further characterization of local tumor extension, particularly when detailed assessment of soft-tissue or aerodigestive tract involvement is required. ^18^F-FDG PET/CT is particularly useful in patients with high-risk differentiated thyroid carcinoma and elevated serum thyroglobulin, especially when radioiodine imaging is negative or when aggressive tumor biology is suspected. It can help identify metabolically active recurrent or metastatic disease and may also be useful for prognostic assessment and evaluation of treatment response in patients with invasive disease. Thus, MRI and ^18^F-FDG PET/CT can complement CT and ultrasound by providing additional anatomic and functional information relevant to staging and treatment planning [14,15].

Histopathological examination remains essential for confirming recurrent thyroid carcinoma and characterizing tumor differentiation. In cases with aggressive or invasive disease, histopathology, supplemented when appropriate by immunohistochemistry, can help distinguish conventional differentiated papillary thyroid carcinoma from poorly differentiated or anaplastic transformation and may provide important information regarding tumor biology. In the present case, histopathological examination of the resected tumor and venous thrombus confirmed recurrent papillary thyroid carcinoma with intravascular tumor thrombosis. Serum thyroglobulin is also an important biomarker for post-treatment surveillance in differentiated thyroid carcinoma, particularly after total thyroidectomy and radioactive iodine therapy. Rising or persistently detectable thyroglobulin may indicate residual or recurrent disease and should be interpreted together with anti-thyroglobulin antibodies and appropriate imaging findings. In the present case, the markedly elevated preoperative serum thyroglobulin supported the presence of recurrent disease, while its subsequent undetectable level after surgery and radioactive iodine therapy was consistent with an excellent early biochemical response [16,17].

Although long-term outcomes remain uncertain because of the rarity of this condition, this case supports previous reports suggesting that aggressive surgical management may be appropriate in carefully selected patients with recurrent PTC and major venous involvement when complete resection is technically feasible. For patients with unresectable or surgically non-amenable recurrent disease, treatment options depend on radioiodine avidity, disease progression, symptoms, and molecular characteristics. Radioactive iodine therapy remains an option for radioiodine-avid disease, whereas external-beam radiotherapy or other local therapies may be considered for selected patients with unresectable locoregional disease. In patients with progressive, symptomatic, or threatening radioiodine-refractory disease, systemic therapy, including multikinase inhibitors such as lenvatinib or sorafenib and molecularly targeted therapies when actionable alterations are identified, may be considered. These approaches should be individualized within a multidisciplinary treatment strategy [18,19]

## Conclusion

Intravascular tumor thrombus in papillary thyroid carcinoma is a rare but clinically important manifestation of aggressive disease. This case highlights an unusual recurrence involving the brachiocephalic vein with retrosternal extension, emphasizing the value of detailed cross-sectional imaging for diagnosis and surgical planning. Despite extensive disease, complete surgical resection with adjuvant radioactive iodine therapy achieved satisfactory short-term disease control, underscoring the role of aggressive multidisciplinary management in selected patients.

## Patient consent

The patient provided written informed consent for the publication of this case report, including all clinical data and accompanying images.

## References

[1] Laurin B.J., Ballard R., Malik I., Mitchell J. (2025). Internal jugular vein tumor thrombus in papillary thyroid cancer: our institution’s experience and a systematic review of the literature. Front Endocrinol (Lausanne).

[2] Kawano F., Tomita M., Tanaka H., Nagahama H., Tashiro K., Nakao H. (2016). Thyroid carcinoma with extensive tumor thrombus in the superior vena cava: a case report. Int J Surg Case Rep.

[3] Póvoa A.A., Teixeira E., Bella-Cueto M.R., Melo M., Oliveira M.J., Sobrinho-Simões M. (2020). Clinicopathological features as prognostic predictors of poor outcome in papillary thyroid carcinoma. Cancers (Basel).

[4] Li M., Trivedi N., Dai C., Mao R., Wang Y., Ning Y. (2019). Does T stage affect prognosis in patients with stage IVB differentiated thyroid cancer?. Endocr Pract.

[5] Xu M., Xi Z., Zhao Q., Yang W., Tan J., Yi P. (2023). Causal inference between aggressive extrathyroidal extension and survival in papillary thyroid cancer: a propensity score matching and weighting analysis. Front Endocrinol (Lausanne).

[6] Pawa R.G., AvantSa R. (2022). Vascular invasion of papillary thyroid carcinoma into neck veins and superior vena cava: a case report. J Clin Diagn Res.

[7] Baberwal P., Asopa R., Basu S. (2024). A rare case of papillary thyroid carcinoma with tumor thrombus extending into the right ventricle. World J Nucl Med.

[8] Adnan Z., Sabo E., Kassem S. (2025). Metastatic papillary thyroid carcinoma with internal jugular vein tumor thrombus: a case report and review of the literature. Front Endocrinol (Lausanne).

[9] Abuduwaili M., Su A., Xing Z., Xia B., Wu Z., Fei Y. (2023). Clinical significance of extrathyroidal extension to major vessels in papillary thyroid carcinoma. J Endocrinol Invest.

[10] Babu S., Salih S., Kumar V., Satheeshan B. (2012). Intravenous tumour thrombus from papillary carcinoma of thyroid: a case report and review of literature. Indian J Surg Oncol.

[11] Koike E., Yamashita H., Watanabe S., Noguchi S. (2002). Brachiocephalic vein thrombus of papillary thyroid cancer: report of a case. Surg Today.

[12] Wada N., Masudo K., Hirakawa S., Woo T., Arai H., Suganuma N. (2009). Superior vena cava (SVC) reconstruction using autologous tissue in two cases of differentiated thyroid carcinoma presenting with SVC syndrome. World J Surg Oncol.

[13] Chung D.J., Krieger D., Moreno N., Renshaw A.A., Alonso R., Cava R. (2019). Papillary thyroid carcinoma: the first case of direct tumor extension into the left innominate vein managed with a single operative approach. World J Endocr Surg.

[14] Ringel M.D., Sosa J.A., Baloch Z., Bischoff L., Bloom G., Brent G.A. (2025). 2025 American Thyroid Association management guidelines for adult patients with differentiated thyroid cancer. Thyroid.

[15] Lal G., Fairchild T., Howe J.R., Weigel R.J., Sugg S.L., Menda Y. (2010). PET-CT scans in recurrent or persistent differentiated thyroid cancer: is there added utility beyond conventional imaging?. Surgery.

[16] Volante M., Lam A.K., Papotti M., Tallini G. (2021). Molecular pathology of poorly differentiated and anaplastic thyroid cancer: what do pathologists need to know?. Endocr Pathol.

[17] Santhanam P., Ladenson P.W. (2019). Surveillance for differentiated thyroid cancer recurrence. Endocrinol Metab Clin North Am.

[18] Kiess A.P., Agrawal N., Brierley J.D., Duvvuri U., Ferris R.L., Genden E. (2016). External-beam radiotherapy for differentiated thyroid cancer locoregional control: a statement of the American Head and Neck Society. Head Neck.

[19] Fleeman N., Houten R., Chaplin M., Beale S., Boland A., Dundar Y. (2019). A systematic review of lenvatinib and sorafenib for treating progressive, locally advanced or metastatic, differentiated thyroid cancer after treatment with radioactive iodine. BMC Cancer.

